# Analysis of Pregnancy Outcomes Using the New IADPSG Recommendation Compared with the Carpenter and Coustan Criteria in an Area with a Low Prevalence of Gestational Diabetes

**DOI:** 10.1155/2013/248121

**Published:** 2013-01-10

**Authors:** Katrien Benhalima, Myriam Hanssens, Roland Devlieger, Johan Verhaeghe, Chantal Mathieu

**Affiliations:** ^1^Department of Endocrinology, UZ Leuven and KU Leuven, Herestraat 49, 3000 Leuven, Belgium; ^2^Department of Obstetrics and Gynecology, UZ Leuven and KU Leuven, Herestraat 49, 3000 Leuven, Belgium

## Abstract

*Aims*. This paper aims to evaluate characteristics and pregnancy outcomes in women prior classified normal by Carpenter and Coustan criteria (old criteria) and now gestational diabetes (GDM) by the IADPSG criteria. *Methods*. Retrospective analysis of 6727 pregnancies is used. Using the old criteria, 222 had GDM (old GDM). Using the IADPSG criteria, 382 had GDM of which 160 had a normal glucose tolerance with the old criteria (new GDM). We compared the new GDM group with the old GDM group and women with normal glucose tolerance with both criteria (NGT group, 6345). *Results*. New GDM women were younger (31.6 ± 4.7 versus 33.3 ± 7.2 years, *P* = 0.010) than old GDM women. Caesarean section was performed in 30.5% of new GDM, in 32.4% of old GDM (*P* = 0.706), and in 23.3% of NGT women (*P* = 0.001). Large for gestational age occurred in 10.8% of new GDM, in 13.8% of old GDM (*P* = 0.473), and in 9.0% of NGT women (*P* = 0.099). Shoulder dystocia occurred in 3.9% of new GDM, in 3.2% of old GDM (*P* = 0.736), and in 1.4% of NGT women (*P* = 0.007). *Conclusion*. Using the IADPSG criteria, more women are identified as having GDM, and these women carry an increased risk for adverse gestational outcome compared to women without GDM.

## 1. Introduction

Gestational diabetes (GDM) is a frequent medical condition during pregnancy and is defined as “any degree of glucose intolerance with onset or first recognition during pregnancy” [1]. Depending on the population in question and on the diagnostic criteria used, GDM prevalence is considered to be between 3% and 14% of all pregnancies [1]. GDM is associated with an increased risk of complications for both the mother and the baby during pregnancy and birth. GDM has long been known to raise the risk of a large for gestational age (LGA) fetus, which in turn increases risks of shoulder dystocia and caesarian deliveries. Short-term risks for the baby include neonatal hypoglycaemia, hyperbilirubinemia, hypocalcaemia, respiratory distress syndrome, and polycythaemia [2, 3]. Shortly after delivery, glucose homeostasis is generally restored to normal, but women with GDM are at high risk of developing type 2 diabetes (T2DM) [4]. There is lack of international uniformity in the approach to screening and diagnosis of GDM. The O'Sullivan and Mahan criteria for diagnosing GDM were not designed to identify women at greater risk of adverse perinatal outcomes but to identify those at higher risk to develop T2DM after pregnancy [5]. Other approaches such as the Carpenter and Coustan criteria rely on standard deviations or percentiles for normal pregnancies [6, 7]. Meanwhile, progressively more data emerge that show that the risk of adverse perinatal outcomes is also associated with degrees of hyperglycaemia less severe than overt diabetes during pregnancy. A milestone study in this respect was the “Hyperglycemia and Adverse Pregnancy Outcomes” (HAPO) study which showed a continuous and graded relationship between the maternal hyperglycaemia and the risk for an adverse perinatal outcome, independent of other risk factors [2]. Based on these results and other studies, the International Association of Diabetes and Pregnancy Study Groups (IADPSG) issued a consensus statement on new criteria for the diagnosis of GDM [8]. However, internationally, there still is a lot of controversy. The American Diabetes Association (ADA) has adopted the IADPSG recommendation since December 2010, while the American College of Obstetricians and Gynecologists (ACOG) advises to continue with the two-step screening strategy [9, 10]. To verify the potential clinical impact of the IADPSG recommendations, we evaluated the clinical characteristics and pregnancy outcomes in women prior classified normal by Carpenter and Coustan criteria and now GDM according to the new IADPSG recommendations.

## 2. Subjects and Methods

The patients of this retrospective study were pregnant women attending routine antenatal clinics at the University Hospital Gasthuisberg, UZ Leuven, in Belgium. Approximately, 2200 women are delivered annually at this hospital. The background prevalence of T2DM in Belgium is 5.3% [11]. Belgium has a population of nearly 11 million of which 12% are from an ethnic minority background. In the general adult population, 28% of women are overweight and 13% are obese [12]. Leuven is a medium size city in the region of Flanders with nearly 100 000 inhabitants. Flanders is one of the most urbanized regions in Europe. Health insurance is compulsory. At the start of this century, Flanders had the lowest rate of mothers aged 35 or more (10.9%) and one of the lowest rates of teenage pregnancies (2.2%) among 16 regions in Western Europe [13]. Leuven has a population with a rather low background number of women from ethnic minorities (10.9%), especially when compared to two of largest cities in Belgium, Brussels (28.1%) and Antwerp (14.6%) [12]. In Flanders, screening for GDM is frequently performed in primary care with the nonfasting 50 g glucose challenge test (GCT). Depending on the preferences and customs of both patients and primary care physicians and/or the obstetricians in our center, patients were screened with the GCT in primary care or at the hospital. There are no directives in our center that women at a higher risk of adverse pregnancy outcomes or under more intensive hospital followup should not be screened in primary care. However, due to inconsistent recording in the electronic medical records in our hospital of both the results of screening as the general characteristics of women who received screening in primary care, only women who received screening for GDM in our hospital were included in our study. Women are not yet universally screened for pregestational diabetes early in pregnancy in our hospital. Form 2005 to 2010, 53.0% of all 12699 pregnancies received screening for GDM in our hospital. We therefore retrospectively analyzed 6727 pregnancies who received screening for GDM. All pregnant women were screened and diagnosed according to the Fifth International Workshop Conference criteria [14]. Women received a GCT, and those testing positive (threshold after 1 h ≥ 140 mg/dL) had a 3 h 100 g oral glucose tolerance test (OGTT) at 24–28 gestational weeks (normal values: 0 min < 95 mg/dL; 60 min < 180 mg/dL; 120 min < 155 mg/dL; 180 min < 140 mg/dL). Women with GDM were treated with insulin 1-2 weeks after the implementation of dietary measures when fasting plasma glucose (FPG) >90 mg/dL and/or 2 h postprandial glycaemia >120 mg/dL. Of all women with GDM, 27% received insulin. Women negative on the GCT or the OGTT were considered to have a normal glucose tolerance (NGT). The prevalence of GDM using the Carpenter and Coustan criteria (old criteria) was 3.3%. Using the new IADPSG criteria (for a 2 h 75 g OGTT, normal values: 0 min < 92 mg/dL; 60 min < 180 mg/dL; 120 min < 153 mg/dL; diagnosis of GDM if one or more values exceeded the threshold), the prevalence of GDM increased to 5.7%. Using the old criteria, 222 women had GDM (old GDM group). Using the IADPSG criteria, 382 had GDM of which 160 had an NGT with the old criteria (new GDM group). The new GDM group was not treated as considered normal using the Carpenter and Coustan criteria. 86.3% (138) of women in the new GDM group had one value of plasma glucose under the 100 g OGTT higher than the Carpenter and Coustan criteria. 

Clinical characteristics and pregnancy outcomes were compared between the new GDM group, the old GDM group, and the NGT group (normal glucose tolerance with both criteria, 6345). 

*Outcomes *were obtained from review of the electronic database.Maternal characteristics recorded were age, body mass index (BMI at first prenatal visit), ethnicity, and parity. Maternal outcomes recorded were overweight (BMI ≥ 25 kg/m²), obesity (BMI ≥ 30 kg/m²), gestational hypertension (blood pressure ≥140/90 mmHg), preeclampsia (hypertension + proteinuria or in combination with reduced growth or HELPP-syndrome), preterm delivery (defined as <37 weeks of gestation), and caesarean section (planned + emergency sections combined). Neonatal outcomes recorded were macrosomia (birth weight >4 kg), LGA (birth weight >90 percentile according to validated Flemish growth charts adjusted for sex and parity), shoulder dystocia, Apgar score (at one and five minutes), admission at the neonatal intensive care unit (NICU), and intrauterine growth retardation (IUGR) [15].

HbA1c was measured by reversed-phase cation-exchange chromatography (ADAMS HA-8160, Menarini Diagnostics Benelux, Zaventem, Belgium). Plasma glucose was measured by an automated colorimetric-enzymatic method (hexokinase-glucose-6-phosphate-dehydrogenase, application 668) on a Hitachi/Roche-Modular P analyzer.

### 2.1. Statistical Methods

Statistical analyses were performed using SPSS 19.0. Continuous variables (normally distributed) are expressed as mean (SD) and categorical data expressed as percentage. To compare variables between the different groups independent samples, *t*-tests were used for normally distributed continuous variables and chi-squared tests for categorical variables. Logistic regression was used to analyse the impact on outcomes of possible confounders such as maternal age, parity, and ethnicity. A *P* value of <0.05 (two-tailed) was considered significant. 

## 3. Results

Of the 6727 women studied, mean age was 31.0 ± 4.9 years, and mean BMI was 23.7 ± 4.4 kg/m² in the total study population. Of all women, 30.7% were overweight and 8.1% were obese. Also, 89.9% of women were of Caucasian descent and 10.1% were from black or minority ethnic origin (BME). The largest BME group was of North African descent (3.9% of all women). Of all women, 16.6% were multiparous and 38.3% were primigravid. 

Using the Carpenter and Coustan criteria, 222 women had GDM (old GDM group). Using the IADPSG criteria, 382 had GDM of which 160 had an NGT with the old criteria (new GDM group). Women from the new GDM group were significantly younger (31.6 ± 4.7 versus 33.3 ± 7.2 years, *P* = 0.010) and had a similar BMI (23.3 ± 3.7 kg/m² versus 24.1 ± 4.5 kg/m², *P* = 0.281) other than the old GDM group. Both the new GDM and old GDM groups were significantly more often from a BME origin (17.4% and 19.7% versus 9.5%, *P* < 0.001) and more often multiparous (25.5% and 20.6% versus 16.3%; only significantly different for the new GDM group) than the NGT group (Table 1). 

All oral glucose tolerance tests results were significantly higher in the new GDM group than in the NGT group and were significantly lower than in the old GDM group (Table 2). Mean Hba1c at diagnosis in the old GDM group was 37 mmol/mol (5.5% ± 0.5%). At diagnosis of GDM by the Carpenter and Coustan criteria, 4.2% of women had a mean Hba1c ≥ 48 mmol/mol (6.5%).

There were no significant in-between group (new GDM, old GDM and NGT) differences in the rate of gestational hypertension, preeclampsia, preterm delivery, abnormal Apgar scores, and admission on the NICU (Table 3). These results were not different when adjusted for confounding factors such as maternal age, parity, and ethnicity. The rate of IUGR was low and not significantly different between the groups (0.4% in the new GDM group, 0.5% in the old GDM group, and 1.9% in the NGT group). The rate of delivery by caesarean section was similarly high in the new GDM group as in the old GDM group (30.5% versus 32.4%; *P* = 0.706), and it was significantly higher than in the NGT group (23.3%, *P* = 0.001). Maternal age (*P* < 0.001) and the rate of women from a BME (*P* = 0.029) origin were significant predictors of the lower rate of delivery by caesarean section in the NGT group compared to the new GDM group. Mean birth weight was not significantly different between the three groups (3278.4 ± 663.3 g in the new GDM group, 3306.7 ± 622.1 g in the old GDM group, and 3310.9 ± 596.3 g in the NGT group). The rate of macrosomia was also not significantly different between the three groups: 8.5% in the new GDM group, 10.1% in the old GDM group, and 9.1% in NGT group. The rate of LGA was only significantly higher in the old GDM group compared to the NGT group (10.8% in the new GDM group, 13.8% in the old GDM group, and 9.0% in NGT group). The rate of shoulder dystocia was similarly high in the new GDM group (3.9%) as in the old GDM group (3.2%, *P* = 0.736), and this was significantly higher than in the NGT group (1.4%, *P* = 0.007). Moreover 36.1% of women with GDM diagnosed by the IADPSG criteria had an FPG meeting the threshold for GDM. Hence, 28.0% (45) of the new GDM group had an elevated FPG. The rate of shoulder dystocia remained significantly higher compared to the NGT group when only the subgroup of the new GDM group with elevated FPG was used for analyses (*P* < 0.001). These results were also not different when adjusted for confounding factors such as maternal age, parity, and ethnicity. Besides a higher rate of BME origin in the new GDM group with an elevated FPG (56.3% versus 10.9%, *P* = 0.004), characteristics and pregnancy outcomes were the same as the other patients in the new GDM group.

## 4. Discussion

The IADPSG criteria identified 160 new cases as GDM, previously considered as normal using the Carpenter and Coustan criteria. This new group of GDM was significantly younger compared to the GDM group diagnosed by the Carpenter and Coustan criteria, and it was significantly more often from a BME origin and multiparous than the NGT group. The women in the new GDM group had not been treated and had worse pregnancy outcomes than the NGT group; rates of caesarian section and shoulder dystocia were significantly higher compared to the NGT group but not significantly higher than the old GDM group. Our findings suggest that glucose concentrations lower than those utilized to diagnose GDM in the past correlate with clinically important perinatal complications, confirming the HAPO study results [2]. Two large randomized intervention trials have demonstrated improvement in perinatal outcomes in the group of women who received treatment of mild glucose intolerance during pregnancy, especially in the frequency of LGA [16, 17]. These studies also correlate maternal hyperglycaemia below the threshold for overt diabetes with clinically important perinatal complications reducible with treatment.

In our study, the rate of macrosomia was not higher in the new GDM group compared to the other groups. The rate of LGA was highest in the old GDM group, and this was only significantly different compared to the NGT group. The rate of LGA in the new GDM group was higher compared to the NGT group, but this did not reach statistical significance. This is in line with a recent Italian study which also showed a nonsignificant higher rate of LGA compared to the NGT group in women reclassified GDM by IADPSG criteria but considered normal using the Carpenter and Coustan criteria on a 100 g OGTT [18]. However, they did show a significantly higher ponderal index for the newborn in the reclassified group compared to both the NGT group and GDM group based on the Carpenter and Coustan criteria. The ponderal index of the newborn might have been a more accurate measurement, but due to lack of data on the length of the newborn in our database, we could not calculate the ponderal index in our study. Despite similar rates of LGA in the new GDM group and NGT group in our study, rates of caesarian section and shoulder dystocia were significantly higher in the new GDM group than in the NGT group. Other anthropometric measures, including measurement of the newborn's fat mass, which was not available in our database, have clearly been shown to be a more accurate measurement of fetal overgrowth in the HAPO study [19]. A recent Mexican study showed that GDM prevalence increased almost threefold using the IADPSG criteria compared to the former ADA criteria using a 100 g OGTT, but there was no significant difference in the prevalence of LGA newborns [20]. As in our study, they also describe a low prevalence of LGA similar to normal pregnant women (7.4% in the IADPSG group, 6.0% in the ADA group, and 5.8% in the NGT group). The rather low rate of LGA seen in our study in all three groups might be related to the rather low percentage of women with overweight or obesity in our cohort since there is convincing evidence that pregestational maternal weight and/or weight gain are likely to play an important role in the development of LGA. The Atlantic Diabetes in Pregnancy study (DIP), on the other hand, showed that the prevalence of GDM based on the IADPSG criteria when compared with WHO criteria increased from 9.4% to 12.4% [21]. Women who were classified as NGT by WHO criteria but as having GDM by IADPSG criteria also had significant adverse pregnancy outcomes compared with IADPSG-defined NGT, including increased rates of LGA (26.8% versus 16.2%). In our study, the rate of overweight women was not significantly different between the three groups. In the HAPO study, in absolute numbers, the majority of LGA was found in the low risk groups, simply because these groups had the highest numbers of women [2]. Controlling hyperglycaemia in pregnancy does not seem to be the only essential factor to decrease the problem of LGA newborns. This is confirmed by recent analyses of the HAPO study showing that both maternal GDM and obesity are independently associated with adverse pregnancy outcomes and that the combination of obesity and GDM shows a greater risk of adverse pregnancy outcomes than either obesity or GDM alone [19]. It was also shown that maternal BMI was a stronger predictor for LGA than maternal glucose in all except the highest glucose category in the HAPO study [22]. Another reason for the lower rate of LGA in the new GDM group compared to the old GDM group might be that by using the IADPSG criteria, women with milder GDM are detected, and therefore lower rates of LGA could be expected. 

Using the IADPSG criteria, prevalence of GDM increased from 3.3% to 5.7% in our study. Compared to diagnosis with the Carpenter and Coustan criteria, this led to a relative increase of 72.0% in the number of women diagnosed with GDM. The combined prevalence of GDM was 17.8% in the HAPO study, but analysis of the frequency of GDM at the different centers showed that the prevalence of GDM varied substantially among sites, with the lowest prevalence of GDM in Israel (9.3%) and the highest prevalence of GDM in the US (Bellflower, CA) with 25.5% [23]. In the HAPO study, overall, 55% had an FPG meeting the threshold for GDM, but there was also an important variation between the different centers in the relative diagnostic importance of fasting, 1 h and 2 h glucose levels [23]. The reasons for the differences are not clear but may relate to the difference in the frequency of obesity and degree of abnormal glucose metabolism in the general populations. In our study, 36.1% of GDM based on the IADPSG criteria had an FPG meeting the threshold for GDM, and in the new GDM group, only 28.0% had an elevated FPG.In contrast, using the IADPSG screening strategy in a large cohort of the United Arab Emirates, FPG independently could have avoided the OGTT in 50.6% of women [24]. This highlights the need to obtain data on GDM prevalence and data on the glucose measures that fulfill the diagnostic criteria with the IADPSG criteria for our own population as this will impact the strategy used for diagnosis of GDM. In our population, it does not seem reasonable to perform an FPG as an initial step, reserving a full OGTT for those with nondiagnostic FPG.

In our study, 4.2% of women in the old GDM group had an Hba1c diagnostic of diabetes, which highlights the importance of screening for pregestational diabetes early in pregnancy as these pregnancies are at an increased risk for congenital malformations. This has now also been recommended by the IADPSG recommendation [8]. 

The strength of our study includes the analysis of a relatively large cohort of pregnant women using a good database analyzing a large number of maternal characteristics and pregnancy outcomes. A limit of our study is the retrospective nature of the analysis and the lack of data on maternal weight gain during pregnancy and lack of data on the ponderal index of the newborn. Because the population attending our university center is a population with a rather low background number of women from ethnic minorities, especially when compared to the largest cities in Belgium, the results of our study cannot be extrapolated to the general Belgium population. Due to our current two-step screening strategy, a substantial number of women with a high FPG in the first trimester or at the time of screening for GDM in the second trimester might have been missed. The prevalence of GDM based on the IADPSG criteria is probably an underestimation in our study since it is known that by using a two-step screening strategy with a GCT based on a threshold of 140 mg/dL, a substantial number of women with GDM might have been missed since it has been shown that around 10% of women with GDM have a GCT <140 mg/dL (1). Another limit is that comparisons were drawn on different sets of criteria. The 1-hour and 2-hour tests could therefore be higher than if a 75 g OGTT would have been used. The new GDM group might therefore include women without GDM by the IADPSG criteria if a 75 g OGTT would have been used. On the other hand, the previous ADA recommendations, did recommend that the Carpenter and Coustan criteria could be used both with the 100 g OGTT as with the 75 g OGTT (1). To address this, characteristics and pregnancy outcomes were analyzed in the new GDM group with an elevated FPG compared to the other patients in the new GDM group, and analyses did not differ significantly expect for a higher rate of BME origin in the subgroup of new GDM with an increased FPG. In particular, the rate of shoulder dystocia remained significantly higher in the new GDM group with an elevated FPG compared to the NGT group.

In conclusion, we show that women prior classified normal by Carpenter and Coustan criteria and now GDM by the new IADPSG criteria are different in phenotype; they are younger compared to the old GDM group and more often from a BME origin and multiparous than the NGT group. Moreover, these women do have an impaired gestational outcome compared to the NGT women. They have increased rates of caesarean section and shoulders dystocia compared to the NGT group despite similar rates of LGA. Further studies are necessary on the consequences of the new IADPSG criteria on the frequency and outcomes of GDM. Data form randomized clinical trials using the IADPSG criteria are necessary since the expected benefit on pregnancy outcomes using the new IADPSG recommendations are mostly based on observational data and extrapolation from the two large intervention studies. We also need more data on the cost effectiveness of such screening strategy, especially in populations with a rather low background prevalence of GDM as seen in our study. It is currently also not clear what the risk is for women with GDM diagnosed through the IADPSG criteria, for the development of T2DM postpartum.

## Figures and Tables

**Table 1:** Differences in clinical characteristics between the new GDM group, the old GDM group, and NGT group.

	New GDM Group 1	*P* 1 versus 2	Old GDM Group 2	*P* 2 versus 3	NGT Group 3	*P* 1 versus 3
*N* (%)	160 (2.4)		222 (3.3)		6345 (94.3)	
Age (SD)	31.6 (4.7)	0.010	33.3 (7.2)	<0.001	30.9 (4.8)	0.103
BMI (SD)	23.3 (3.7)	0.281	24.1 (4.5)	0.426	23.7 (4.4)	0.510
BMI ≥ 25 kg/m²	27.5%	0.430	35.1%	0.477	30.6%	0.701
BME	17.4%	0.677	19.7%	<0.001	9.5%	<0.001
Multiparous	25.5%	0.272	20.6%	0.109	16.3%	0.003
Primigravid	38.2%	0.309	43.2%	0.102	38.1%	0.276

GDM: gestational diabetes; new GDM group: prior classified normal by old criteria and now GDM according to the new IADPSG criteria; old GDM group: classified GDM according to Carpenter and Coustan criteria (old criteria); NGT: normal glucose tolerance; SD: standard deviation; BME: black or minority ethnic origin.

**Table 2:** Differences in oral glucose tolerance tests results between the new GDM group, the old GDM group, and NGT group.

Mean plasma glucose mg/dL (SD)	New GDM Group 1	*P* 1 versus 2	Old GDM Group 2	*P* 2 versus 3	NGT Group 3	*P* 1 versus 3
*N* (%)	160 (2.4)		222 (3.3)		6345 (94.3)	
0 min	85.6 (10.8)	<0.001	92.0 (18.1)	<0.001	77.8 (6.1)	<0.001
60 min	164.2 (28.0)	<0.001	191.3 (32.8)	<0.001	133.9 (23.3)	<0.001
120 min	147.1 (25.9)	<0.001	180.1 (31.2)	<0.001	119.6 (19.8)	<0.001

GDM: gestational diabetes; new GDM group: prior classified normal by old criteria and now GDM according to the new IADPSG criteria; old GDM group: classified GDM according to Carpenter and Coustan criteria (old criteria); NGT: normal glucose tolerance; SD: standard deviation.

**Table 3:** Differences in pregnancy outcomes between the new GDM group, the old GDM group, and NGT group.

	New GDM Group 1	*P* 1 versus 2	Old GDM Group 2	*P* 2 versus 3	NGT Group 3	*P* 1 versus 3
Gestational hypertension	1.9%	0.217	4.3%	0.483	3.4%	0.474
Preeclampsia	0.6%	0.825	0.5%	0.812	0.6%	0.969
Preterm delivery	29.2%	0.099	21.6%	0.240	25.9%	0.241
Caesarean section	30.5%	0.706	32.4%	0.002	23.3%	0.001
Macrosomia	8.5%	0.607	10.1%	0.600	9.1%	0.848
LGA	10.8%	0.473	13.8%	0.036	9.0%	0.099
Shoulder dystocia	3.9%	0.736	3.2%	0.029	1.4%	0.007
Apgar score <7 at 5 min	2.6%	0.662	1.9%	0.704	1.7%	0.625
Admission NICU	12.0%	0.448	14.6%	0.101	10.9%	0.261

GDM: gestational diabetes; new GDM group: prior classified normal by old criteria and now GDM according to the new IADPSG criteria; old GDM group: classified GDM according to Carpenter and Coustan criteria (old criteria); NGT: normal glucose tolerance; LGA: large for gestational age; NICU: neonatal intensive care unit.
